# Supplementary material for: Content-rich biological network constructed by mining PubMed abstracts
Source: BMC Bioinformatics. 2004 Oct 8;5:147. doi: 10.1186/1471-2105-5-147 (PMC528731; doi:10.1186/1471-2105-5-147)
Supplement: Additional File 2 — The original results of the above study (non-essential files are deleted to keep the file size under the limit set by BMC bioinformatics). [file 1471-2105-5-147-S2.bz2 › chilibotAdditionalFile2/dip10/613ID10558980E10996/html/UBE3A_UBE2L3.html]

 


 **UBE3A** and **UBE2L3** 
  
Found 61 abstracts in PubMed, retrieved 10.  
 

 What does Google say? 
 PDF only 
| .edu only 

---

**Interactive relationship** (e.g. stimulation, inhibition, etc)

**Neutral relationship**- **UBE3A**  encodes  **UBE3A**  E6 AP  [ **UBE3A** ] , a ubiquitin protein ligase  [ **UBE2L3** ] , and shows brain specific imprinting, with brain expression predominantly from the maternal allele.  Ref: 11895368 Neurobiol Dis, 2002
- also called ubiquitin protein ligase  [ **UBE2L3** ]  E3A  [ **UBE3A** ]  or  **UBE3A** , which functions as an ubiquitin protein ligase  [ **UBE2L3** ] .  Ref: 12619117 Hum Mutat, 2003

**Non-interactive relationship** (e.g. studied together, co-existance, homology, etc.)

- We identified a number of positive interacting clones that encode ubiquitin protein ligase  [ **UBE2L3** ]  E3A  [ **UBE3A** ]   **UBE3A** .  Ref: 12207887 Biochem Biophys Res Commun, 2002
- It results from a maternal deletion of human chromosome 15q11 13 containing two candidate genes E6 P ubiquitin protein ligase  [ **UBE2L3** ]   **UBE3A**  and GABA A receptor beta3 subunit GABRB3, the latter of which has been also linked to autism.  Ref: 12672542 Neurosci Lett, 2003
- On the basis of discoveries made in molecular biology, patients can be classified as belonging to five types deletion, paternal uniparental disomy UPD, imprinting defects, mutation of the  **UBE3A**  ubiquitin protein ligase  [ **UBE2L3** ]  gene and unidentified mechanism 15% 20% of patients.  Ref: 12373673 Rev Neurol, 2002
- Typical NEDD4 orthologues that contain a C2 Ca 2 lipid binding and a HECT Homologous to the E6 AP  [ **UBE3A** ]  Carboxyl Terminus ubiquitin protein ligase  [ **UBE2L3** ]  domain, and multiple WW domains have been shown to regulate the epithelial sodium channel ENaC.  Ref: 11840194 Eur J Hum Genet, 2001
- One candidate is the ubiquitin protein ligase  [ **UBE2L3** ]  gene  **UBE3A** , which is maternally expressed only and localized to the 15q region.  Ref: 11489053 J Intellect Disabil Res, 2001
- 4 a mutation in the E3 ubiquitin protein ligase  [ **UBE2L3** ]  gene  **UBE3A** .  Ref: 11748306 J Med Genet, 2001
- VCY2 protein interacts with the HECT domain of ubiquitin protein ligase  [ **UBE2L3** ]  E3A  [ **UBE3A** ] .  Ref: 12207887 Biochem Biophys Res Commun, 2002
